# Can Botulinum Toxin A Still Have a Role in Treatment of Lower Urinary Tract Symptoms/Benign Prostatic Hyperplasia Through Inhibition of Chronic Prostatic Inflammation?

**DOI:** 10.3390/toxins11090547

**Published:** 2019-09-19

**Authors:** Bing-Juin Chiang, Hann-Chorng Kuo, Chun-Hou Liao

**Affiliations:** 1 College of Medicine, Fu-Jen Catholic University, New Taipei City 24205, Taiwan; bingjuinchiang@gmail.com; 2 Department of Urology, Cardinal Tien Hospital, New Taipei City 23148, Taiwan; 3 Department of Life Science, College of Science, National Taiwan Normal University, Taipei 11677, Taiwan; 4 Department of Urology, Buddhist Tzu Chi General Hospital, and Tzu Chi University, Hualien 97002, Taiwan; hck@tzuchi.com.tw; 5 School of Medicine, Tzu Chi University, Hualien 97004, Taiwan

**Keywords:** lower urinary tract symptoms, botulinum toxin, benign prostatic hyperplasia, prostatitis, inflammation

## Abstract

Patients with benign prostatic hyperplasia (BPH) can exhibit various lower urinary tract symptoms (LUTS) owing to bladder outlet obstruction (BOO), prostatic inflammation, and bladder response to BOO. The pathogenesis of BPH involves an imbalance of internal hormones and chronic prostatic inflammation, possibly triggered by prostatic infection, autoimmune responses, neurogenic inflammation, oxidative stress, and autonomic dysfunction. Botulinum toxin A (BoNT-A) is well recognized for its ability to block acetylcholine release at the neuromuscular junction by cleaving synaptosomal-associated proteins. Although current large clinical trials have shown no clinical benefits of BoNT-A for the management of LUTS due to BPH, BoNT-A has demonstrated beneficial effects in certain subsets of BPH patients with LUTS, especially in males with concomitant chronic prostatitis/chronic pelvic pain syndrome and smaller prostate. We conducted a review of published literature in Pubmed, using Botulinum toxin, BPH, BOO, inflammation, LUTS, and prostatitis as the key words. This article reviewed the mechanisms of BPH pathogenesis and anti-inflammatory effects of BoNT-A. The results suggested that to achieve effectiveness, the treatment of BPH with BoNT-A should be tailored according to more detailed clinical information and reliable biomarkers.

## 1. Introduction

Benign prostatic hyperplasia (BPH) is a term exclusively used for describing benign histological patterns in the European Association of Urology (EAU) and the American Urological Association (AUA) guidelines [1,2]. BPH could lead to benign prostatic enlargement (BPE) or benign prostatic obstruction (BPO) resulting in male lower urinary tract symptoms (LUTS). LUTS are grouped into three categories: storage (increased daytime frequency, nocturia, urgency, and urinary incontinence), voiding (hesitancy, slow stream, intermittent stream, straining, and terminal dribble), and post-micturition (post-micturition dribble and feeling of incomplete emptying) symptoms [3]. In addition to BPH, other causes of male LUTS include structural or functional abnormalities of the bladder and its surrounding tissues, and some non-urological conditions [4]. Lower urinary tract dysfunction (LUTD) is defined as signs observed by the physician, including simple means, to verify and quantify symptoms [3]. From a clinical perspective, BPH usually refers to LUTD caused by BPE or BPO.

We conducted a review of published literature in Pubmed, using Botulinum toxin, BPH, bladder outlet obstruction (BOO), inflammation, LUTS, and prostatitis as the key words. We reviewed the mechanisms of BPH pathogenesis and anti-inflammatory effects of BoNT-A. We tried to determine the role of botulinum toxin A (BoNT-A) in treatment of LUTS/BPH.

## 2. Ambiguous Mechanisms of LUTS Related to BPH

Prevalence of LUTS generally increases with age. A large cross-sectional population-based study from the Asia-Pacific region reported LUTS prevalence to be higher in men than women [5]. More men had voiding symptoms (45.3%) than women (31.3%) [5], and the phenomenon could be attributed to BPH. Interestingly, the epidemiologic study showed more storage symptoms (49.9%) in men than voiding symptoms (45.3%). This could be attributed to BOO, which is capable of reducing bladder blood flow and subsequently causes chronic bladder ischemia, as reported by various epidemiologic and clinical studies [6,7,8]. BOO is also associated with repeated episodes of prolonged detrusor ischemia in pigs with an artificially implanted ring around urethra [9]. BOO causes a reduction in acetylcholine esterase staining nerves in detrusor muscle and expression of hypoxia-inducible factor 1 alpha, a cellular marker of hypoxia [10,11]. Moreover, experimentally, BOO has been shown to exhibit elevated cystometric voiding pressure, reduced urine flow rates, generated bladder hyperactivity, and increased bladder detrusor hypertrophy [12,13]. Further, BOO related bladder ischemia also causes denervation supersensitivity, leading to a fundamental reorganization of the detrusor’s electrical activity and C-fiber mediated micturition reflexes [13,14,15,16]. BOO with high bladder pressure could also induces adaptive change of bladder wall, including detrusor muscle hypertrophy, bladder wall fibrosis and reduced bladder compliance [17]. Therefore, besides the preconceived voiding symptoms, BPH also causes varied storage symptoms related to bladder response to BOO. In addition, the prevalence of prostatitis-like symptoms in a community-based study was 11.5% and 8.5% in younger (<50 years) and older (≥50 years) men, respectively [18]. This study also measured irritative and obstructive voiding symptom severity (score, 0 to 10), where men with prostatitis-like symptoms showed significantly higher urinary symptom score. Prostatic inflammation is believed to play an important role in the BPH pathogenesis and progression [19,20]. In brief, most BPH related LUTS arises from BPE/BOO, prostatic inflammation, and bladder response to BPE/BOO. Therefore, it is difficult to distinguish the specific etiology of male LUTS through clinical practice. Although the exact mechanism underlying BPH related LUTS is unknown, its treatment should probably be tailored according to the cause of LUTS.

## 3. Mechanism of BPH Related to BOO

BPH causes obstruction by inducing functional and morphological changes in the prostate. Functionally, an increase in prostatic smooth muscle tone has been confirmed, which is influenced sympathetically [21]. Moreover, the presence of metabolic syndrome is associated with increased sympathetic nervous system activity and LUTS [22,23]. In such cases, alpha(α)-adrenergic antagonists can improve BPH-induced male LUTS [24]. Morphologically, BPH is characterized by unregulated proliferation of connective tissues, smooth muscles, and glandular epithelium [25]. Tissue proliferation leads to increased prostate volume (PV), subsequently compressing the prostatic urethra. McNeal found that BPH patients had an increase in BPH nodules in the periurethral zone and size of glandular nodules [26]. Further, either epithelium or fibro-muscular stroma proliferation could be found in the resected BPH tissues [27,28].

## 4. Mechanism of BPH-Imbalance of Internal Hormones 

Abundant evidence showed that development of BPH requires testicular androgen [29]. Conversion of dihydrotestosterone (DHT), a metabolite of testosterone, in the prostate is considered as a major factor involved in the BPH pathogenesis. Elevated serum DHT level is associated with larger PV and higher prevalence of BPH [30]. Prostatic DHT and androgen receptor (AR) levels increase with age [31], whereas testosterone level declines with age. Increase in estrogen to testosterone ratio has been recognized as an important factor inducing the development of prostatic inflammation and cytokines [32]. In such cases, administration of 5α-reductase type II inhibitor resulted in increased plasma testosterone levels and further reduced the prostatic inflammation, suggesting the protective effect of testosterone against inflammation compared to that of DHT [33,34]. Imbalance in testosterone and estrogen levels also contributes to decreased activity of some suppressor cells, which maintain tolerance to prostatic antigen and prevent autoimmunity [35,36]. Moreover, the insulin-like growth factor (IGF) signaling pathway has been implicated in BPH development [37]. An IGF receptor antagonist, metformin, has an anti-proliferative effect, which attenuated testosterone-induced BPH in rats by decreasing the expression of estrogen receptor alpha [38]. Furthermore, neurotransmitter serotonin (5-HT) could possibly play a role in the pathogenesis of BPH. 5-HT can downregulate ARs and prevent prostate branching [39]. However, 5-HT depletion contributes to BPH development through modulation of ARs [39]. In summary, imbalance of internal hormones results in the development of BPH and prostatic inflammation.

## 5. Mechanism of BPH-Chronic Inflammation 

A previous clinical study showed that men with prostatitis-like symptoms have significantly higher urinary symptom score [18]. Young-onset prostatitis was positively associated with LUTS [40]. Chronic inflammation along with BPH can coexist in human pathologic specimens [41]. Correlations were found between histopathology of chronic inflammation and severity of LUTS in a subgroup of patients from the randomized REDUCE (reduction in the use of corticosteroids in exacerbated COPD) trial [42]. Moreover, nonsteroidal anti-inflammatory drugs were inversely associated with the onset of LUTS [43]. These clinical results suggested the involvement of prostatic inflammation in the pathogenesis of BPH with LUTS.

The most important cytokine involved in the development of BPH is interleukin (IL)-8, which can directly promote epithelial and stromal proliferation [44]. Plasma IL-8 could serve as a reliable surrogate marker of prostatic inflammatory conditions, including chronic prostatitis and BPH [45]. Moreover, IL-8 can induce stromal cells for the emergence of a reactive myofibroblast phenotype [46]. Human BPH cells, including epithelial and stromal cells, act as antigen-presenting cells (APCs), which can secrete IL-8 and associated cytokines [47]. These cytokines promote prostatic immune cells upregulation for more specific cytokines, which in turn recruit more lymphomononuclear cells. These recruited lymphomononuclear cells express cognate receptors, CXCR1 and CXCR2, which induce proliferation of prostatic cells through autocrine/paracrine effect and generation of fibroblast grow factors [48]. Therefore, the cross-talk between the BPH and immune cells creates a positive feedback loop that can amplify inflammation, and the intraprostatic chronic inflammatory processes are induced and sustained.

## 6. Etiology of Prostatic Inflammation 

The etiology of prostatic inflammatory process remains unclear. It is believed that several possible mechanisms are responsible for triggering a prostatic inflammatory response. Firstly, infection-induced inflammation hypothesis is evidenced by the presence of bacterial and viral strains in BPH tissue specimens [49]. Toll-like receptors (TLRs) expressed by BPH cells recognize structurally conserved molecules derived from pathogens. TLRs-mediated production of proinflammatory cytokines (IL-6) and chemokines (IL-8 and CXCL10) initiate and enhance the inflammatory process [50]. Secondly, autoimmune responses could be involved in prostatic inflammation. Epidemiologic studies showed that the prevalence of chronic prostatitis/chronic pelvic pain syndrome (CP/CPPS) is eight times more than bacterial prostatitis [51]. Prostate, as well as testes, are considered as immunologically privileged organs [52]; however, self-antigens release following a tissue injury results in autoimmunity [53]. Autoantibodies against prostate-specific antigen (PSA) or prostate acid phosphatase (PAP) were shown to be involved in the prostatic inflammatory process [54]. PSA has been demonstrated to be able to activate CD4+ T cells [55]. In addition, BPH cells, as well as other APCs, produce high levels of IL-12 and IL-23, promoting CD4+ T cell activation and differentiation [47], which in turn, differentiate into interferon gamma-secreting T-helper (Th) type 1 and IL-17-secreting Th17 cells [48]. These downstream pro-inflammatory cytokines manifest a positive feedback signal to inflammatory interaction between BPH and immune cells. Thirdly, neurogenic inflammation plays an important role in chronic prostatic inflammation. Neurotrophin, a nerve growth factor (NGF), is responsible for mediating prostatic neurogenic inflammation. The serum NGF level was found to be correlated with the severity of pain in CP/CPPS [56]. Moreover, damaged tissue has been found to contain higher NGF level [57], which can cause mast cells degranulation [58]. Infiltrating mast cells in BPH tissues can promote BPH development via activation of IL-6/Signal transducer and activator of transcription 3/Cyclin D1 signaling pathway [59]. NGF can also sensitize sensory nerve and induce production of neuropeptides, including substance P and calcitonin gene-related peptide (CGRP) [60]. Further, substance P can stimulate reactive oxygen species generation via its proinflammatory activity [61]. Prostatitis following intraprostatic formalin injection has been reported to induce prostate-to-bladder afferent cross-sensitization, increased urothelial NGF expression, and subsequent bladder overactivity [62]. Fourthly, oxidative stress can be one of the causes of inflammation. Interestingly, the ARR2PB-Nox4(ARR2PB-NADPH oxidase 4) transgenic mice showed increased prostate weight, increased epithelial proliferation, and histological changes, including epithelial proliferation, stromal thickening, and fibrosis through Nox4 promoting oxidative stress [63].

Furthermore, it is believed that chronic pelvic ischemia can generate oxidative stress [64]. Elderly patients with LUTS showed decreased prostate perfusion on transrectal color Doppler ultrasonography [7]. Serum glutathione peroxidase and superoxide dismutase levels, which have antioxidant effects, reportedly declined in dogs with BPH [65]. Oxidative stress also triggers prostate cells proliferation through the activation of cyclooxygenase (COX) pathways [66,67], whereas COX-2 inhibition can induce significant apoptosis in the prostate cell [67]. Fifthly, the autonomic nervous system (ANS) also contributes to prostatic inflammation and growth. Adrenergic innervation plays a role in prostate growth. ANS hyperactivity is significantly associated with LUTS, and serum norepinephrine level increased after tilt predicted prostate size [68]. Moreover, chronic administration of α1-adrenergic agonists induces proliferation of prostatic cells in a rat model [69]. Besides, norepinephrine can stimulate the proliferation of human non-epithelial prostatic cells [70]. α1-adrenoceptors have been linked with inflammatory pathways through activation of transforming growth factor β signaling cascade, regulating various events associated with the BPH development [69,71]. In summary, the major etiology of chronic prostatic inflammation includes prostatic infection, autoimmune responses, neurogenic inflammation, oxidative stress, and autonomic dysfunction.

## 7. Anti-Inflammatory Effects of Botulinum Toxin A (BoNT-A)

BoNT-A is well-recognized for its ability to block acetylcholine release at the neuromuscular junction by cleaving synaptosomal-associated proteins [72]. Intraprostatic injection of BoNT-A has been shown to induce relaxation of prostatic muscle through downregulation of α-adrenergic receptor expression and reducing smooth muscle contractility [73,74]. On the other hand, BoNT-A also causes morphological atrophy of the glands via chemodenervation and anti-inflammatory effects [75,76]. Clinically, BoNT-A has demonstrated therapeutic anti-inflammatory effects, including the reduction of pain, edema, erythema, and heat emission [77]. A study in a complete Freund’s adjuvant-induced arthritic rat model revealed the anti-inflammatory effect of BoNT-A by attenuating anti-ionized calcium-binding adaptor molecule 1 and IL-1β immune-reactive cells [78]. Moreover, BoNT-A reduces rosacea-associated skin inflammation by directly inhibiting mast cell degranulation [79]. Intravesical BoNT-A injections plus hydrodistension reduce bladder pain and NGF levels in patients with interstitial cystitis [80], probably through blocking bladder pain responses and CGRP release from afferent nerve terminals, as depicted in a rat model [81]. BoNT-A may also inhibit peripheral and subsequent central sensitizations via suppressing substance P, glutamate, and adenosine triphosphate, showing reduction of somatic and visceral pain [82]. Interestingly, BoNT-A pretreatment could inhibit intraprostatic capsaicin injection-induced COX-2 expression in prostate and spinal cord [83]. Furthermore, BoNT-A also significantly prevented oxidative stress in vascular endothelial cells in cutaneous ischemia/reperfusion injured mouse model [84]. It has been shown that BoNT-A inhibits IL-8/CXCR1 signaling cascade in endothelial cells through inhibiting Rho signaling pathways [85]. In summary, BoNT-A exhibits anti-inflammatory effect by suppressing cytokine generation, mast cell activation, neurogenic inflammation, and oxidative stress in different organs. These reports from the basic research of BoNT-A potentially strengthened evidence for its therapeutic effects in patients with BPH; however, further studies are warranted for some of the proven anti-inflammatory effects on prostatic growth and inflammation.

## 8. Clinical Perspectives

Although previous single-arm studies showed promising results in improving international prostate symptom score (IPSS), maximal flow rate (Qmax), PV, and post-void residual urine volume (PVR) following intraprostatic injection of BoNT-A, two recent large scale randomized control trials failed to show significant efficacy of BoNT-A on all outcomes [76,86,87,88,89,90,91,92]. Moreover, a systematic review including three large randomized placebo-control studies (experimental group, *n* = 260; control group, *n* = 262) showed only marginal benefits to IPSS (−1.02; 95% confidence interval: −1.97, −0.07) for the BTX-A versus placebo groups. There were no significant differences in Qmax, PV, and PVR between the two groups, which was attributed to the placebo effect [93]. The EAU guidelines on the management of non-neurogenic male LUTS documented that “Results from clinical trials have shown no clinical benefits for BoNT-A compared to placebo for the management of LUTS due to BPO,” and strongly recommended not to offer intraprostatic BoNT-A injection treatment to patients with male LUTS [1]. Intraprostatic BoNT-A injection is also not listed as one of the treatment choices in the AUA guidelines [2]. However, it is difficult to explain how intraprostatic BoNT-A injection significantly decrease PV, as reported by other meta-analyses [94,95,96].

Although limited reports are available for the therapeutic effects of BoNT-A on CP/CPPS, several beneficial effects on LUTS have been noted. In an uncontrolled randomized clinical trial conducted in men with refractory CP/CPPS, the patients were classified into two groups according to the route of BoNT-A injection, transurethral or transrectal. After intraprostatic injection of BoNT-A (100 U), Qmax, voiding score, and quality of life (QoL) were significantly improved in both groups during the follow-up period [97], with mean initial PV ranging from 36.4 to 37.9 ml among the groups. Another randomized, controlled study of transurethral intraprostatic injection of BTX-A (100 or 200 U depending on PV) showed significant improvement not only in pain score and QoL but also in the urinary domain of chronic prostatitis symptom index 1-month post-injection [98]. Other outcomes, including IPSS, frequency of diurnal, and nocturnal urination, showed significant reduction compared to baseline at 1, 3 and 6-month after BoNT-A injection. The mean initial PV in the experimental group was 22.27 mL. Chuang et al. reported that 16 men with symptomatic BPH and PV <30 mL were successfully treated with transperineal intraprostatic BoNT-A injection [89], with significant improvement in PV and IPSS following BoNT-A injection. However, the mean PV of men included in the two largest randomized trials failed to show beneficial effects on PV (range, 43.8–48.8 mL) [91,92]. Therefore, these results imply that intraprostatic injection of BoNT-A might be effective in relieving LUTS in patients with small prostate and refractory CP/CPPS. As mentioned above, patients suffering from BPH and CP/CPPS might show overlapping symptoms due to similar etiologies shared by BPH and prostatitis.

## 9. Conclusions

Based on the current basic and clinical studies, BoNT-A could still be effective in certain subsets of BPH with LUTS, especially in males with concomitant CP/CPPS and smaller prostate. However, this hypothesis requires further validation through randomized controlled clinical studies. In addition, it is imperative to consider exploring biomarkers of BPH, including NGF level, mast cells/distribution of activated subtypes of immune cells in biopsies, inflammatory-associated cytokines, or ANS dysfunction, as determining predictors of the treatment efficacy. Therefore, for effectiveness, the treatment of BPH with BoNT-A should be tailored according to more detailed clinical information and reliable biomarkers.
